# Automatic segmentation of the core of the acoustic radiation in humans

**DOI:** 10.3389/fneur.2022.934650

**Published:** 2022-09-23

**Authors:** Malin Siegbahn, Cecilia Engmér Berglin, Rodrigo Moreno

**Affiliations:** ^1^Division of Ear, Nose and Throat Diseases, Department of Clinical Science, Intervention and Technology, Karolinska Institute, Stockholm, Sweden; ^2^Medical Unit Ear, Nose, Throat and Hearing, Karolinska University Hospital, Stockholm, Sweden; ^3^Department of Biomedical Engineering and Health Systems, KTH Royal Institute of Technology, Stockholm, Sweden

**Keywords:** acoustic radiation, diffusion MRI, tractography, TractSeg, deep learning

## Abstract

**Introduction:**

Acoustic radiation is one of the most important white matter fiber bundles of the human auditory system. However, segmenting the acoustic radiation is challenging due to its small size and proximity to several larger fiber bundles. TractSeg is a method that uses a neural network to segment some of the major fiber bundles in the brain. This study aims to train TractSeg to segment the core of acoustic radiation.

**Methods:**

We propose a methodology to automatically extract the acoustic radiation from human connectome data, which is both of high quality and high resolution. The segmentation masks generated by TractSeg of nearby fiber bundles are used to steer the generation of valid streamlines through tractography. Only streamlines connecting the Heschl's gyrus and the medial geniculate nucleus were considered. These streamlines are then used to create masks of the core of the acoustic radiation that is used to train the neural network of TractSeg. The trained network is used to automatically segment the acoustic radiation from unseen images.

**Results:**

The trained neural network successfully extracted anatomically plausible masks of the core of the acoustic radiation in human connectome data. We also applied the method to a dataset of 17 patients with unilateral congenital ear canal atresia and 17 age- and gender-paired controls acquired in a clinical setting. The method was able to extract 53/68 acoustic radiation in the dataset acquired with clinical settings. In 14/68 cases, the method generated fragments of the acoustic radiation and completely failed in a single case. The performance of the method on patients and controls was similar.

**Discussion:**

In most cases, it is possible to segment the core of the acoustic radiations even in images acquired with clinical settings in a few seconds using a pre-trained neural network.

## 1. Introduction

The acoustic radiation (AR) is a white matter fiber bundle that connects the Heschl's gyrus (HG) in the cortex with the medial geniculate nucleus (MGN) in the mid-brain (1, 2). The AR is one of the most important fiber bundles of the auditory system (3), and its analysis is relevant for understanding the mechanisms of acoustic stimuli processing and how they are affected by different diseases. For example, diseases such as tinnitus (4, 5), schwannoma (6), and putaminal hemorrhage (7, 8) have been associated with changes in the AR. Reliable methods for extracting the AR are crucial for performing such analyses.

Extracting the AR with tractography from diffusion MRI (dMRI) is challenging (9). First, the AR is a relatively short bundle of approximately 4–6 cm (2), making it especially sensitive to the low resolution of standard imaging acquisitions used in clinics. Second, the AR is very close to other bundles such as the cortico-spinal tract (CST), arcuate fasciculus (AF), the middle longitudinal fasciculus (MLF), the inferior fronto-occipital fasciculus (IFOF), and the optic radiation (OR) (10–12). We have also found that the AR is close to the inferior longitudinal fasciculus (ILF) in some cases. This closeness to other bundles can make it difficult for the tractography method to extract streamlines only related to the AR. Low-resolution dMRI might be unable to disentangle the crossing and kissing fiber bundles from the intersection regions along the AR. This has also been reported as a problem for segmenting neighboring fiber bundles (12). Moreover, MGN, HG, and AR have a large variability among subjects (2, 9, 11, 13).

The fiber bundle connecting the MGN with the HG can be considered the core of the AR. In their review, Maffei et al. (9) discussed that, in addition to the core of the AR, there is evidence from *ex vivo* studies on macaque monkeys that the AR might have extra layers of fibers that create a “belt” that can go beyond the HG and reach the superior temporal gyrus (STG) (14, 15). The core and this belt of the AR are thought to have different functions. The core of the AR might be involved in basic tone processing. In contrast, the belt might be involved in integrating auditory information with other sensory information. Since their purpose is different, neurological and auditory conditions can affect the core and the belt of the AR differently. Thus, having independent segmentation masks for the core and the belt is relevant for further analyses. In this article, we focus on generating segmentation masks of the core of AR.

Different atlases of AR have been proposed in the literature. For example, Bürgel et al. (2) used histology to create a high-resolution atlas of different fiber bundles of the white matter from ten donors, including the AR. More recently, Maffei et al. (16) created an atlas using dMRI acquisitions with ultra-high *b*-values (up to 10,000 *s*/*mm*^2^) and high resolution (1.5 mm isotropic) from the MGH adult diffusion dataset of the human connectome project (HCP) (17, 18). However, as already mentioned, the use of atlases of AR is not ideal due to its reported anatomical variability (1, 2, 9, 16, 19).

Two automatic tools include the segmentation of the AR: XTRACT (20, 21) and TRACULA (22). XTRACT is a tool of the FMRIB Software Library (FSL) (23) that can segment 42 fiber bundles, including the AR. In order to segment the AR, XTRACT runs probabilistic tractography between the HG and the MGN and defines exclusion masks to remove anatomically implausible streamlines. In particular, it uses two coronal planes and an axial plane around the thalamus, a region covering the optic tract and the brainstem as exclusion masks. XTRACT also provides an atlas of the AR based on the HCP young adult dataset (24, 25) and the UK Biobank dataset (26). One potential issue of XTRACT is that its exclusion criteria might be too liberal with respect to knowledge from neuroanatomists (9, 10). Thus, there is a risk that segmentation masks might cover areas that should not be part of the AR.

TRACULA (27) is a tool of FreeSurfer (28) for fiber bundle segmentation. This method uses prior anatomical information of the fiber bundles to steer a Bayesian-based global tractography. The original method included 18 main fiber bundles and did not include the AR. Maffei et al. (22) extended the number of fiber bundles to 42, including the AR. For this, they manually segmented the 42 fiber bundles in 16 subjects of the MGH adult diffusion dataset of the HCP (17, 18).The new definitions were made available in the latest version of FreeSurfer (version 7.2, release date: July 2021).

Regarding the AR, Maffei et al. (22) used a subset of the segmentation masks used by Maffei et al. (16) to create their atlas of AR. One of the issues of TRACULA for segmenting the AR is that the manual dissections in the 16 subjects include too few streamlines. More specifically, the mean number of streamlines extracted per subject in the MGH dataset was 26 (ranging between 2 and 91) for the left side and 32 (ranging between 6 to 70) for the right side. As a comparison, TRACULA uses an average of 1,250 streamlines per subject (ranging between 333 and 2,726) for the left arcuate fascicle. This low number of streamlines used for the AR has the risk of making TRACULA less specific with respect to anatomical variations of the AR. An additional issue of TRACULA is that it uses global tractography, which makes it very time-consuming compared to other methods. Moreover, TRACULA requires the parcellation generated by FreeSurfer, which usually takes several hours.

Wasserthal et al. (29) proposed TractSeg, a method based on artificial intelligence (AI) that is able to segment 72 main fiber bundles from dMRI automatically. The advantages of this method are that it works with standard dMRI acquisitions, even with low *b*-values, is fast (takes a few seconds), does not require a previous registration of images, and, unlike atlases, the results are subject-specific. Due to the aforementioned difficulties in segmenting the AR, the original method did not include the AR. More recently, Wasserthal et al. (29) trained the original neural network using the masks generated by XTRACT (20, 21), including the AR. Thus, since version 2.2. of TractSeg, it is possible to obtain these segmentations with the option “–*tract_definition xtract*”.

Both XTRACT and TRACULA allow the streamlines to go beyond the HG and reach the STG. This means that these methods are not designed to extract the core of the AR. Thus, the main goal of this paper is to assess the possibility of using TractSeg for the segmentation of the core of the AR in datasets acquired in clinical settings.

## 2. Methods

### 2.1. Datasets

We used two datasets in this study. The first one consists of dMRI data from 125 subjects of the HCP young adult dataset (24, 25). A total of 105 of these subjects are exactly the same used by Wasserthal et al. (29) and were used for training the TractSeg (29) models with masks generated using the segmentation methodology proposed in this paper, while the remaining 20 were used for independent testing. The dMRI data of HCP consists of 90 directions for each of the three *b*-values: 1,000, 2,000, and 3,000 *s*/*mm*^2^, and the spatial resolution is 1.25 *mm* isotropic. These images were acquired in Siemens 3T scanners using a spin-echo EPI sequence with a multiband factor of 3, TR/TE is 5,520/89.5 ms, a flip angle of 78 degrees, and a refocusing flip angle of 160 degrees. The images were acquired using a head coil with 32 channels. More details on imaging parameters are available on the website of HCP^1^. The second dataset consists of dMRI data of 34 subjects acquired with the following parameters: isotropic resolution of 2.3 *mm* and 60 directions at *b* = 1,000 *s*/*mm*^2^. The images were acquired at the MRI facility of Karolinska Institute at Karolinska University Hospital in Solna using a GE Discovery 3T MR750 scanner with a spin-echo EPI sequence with TR/TE of 7,000/80.9 ms and flip angle of 90 degrees. The images were acquired using a head coil with 8 channels. The cohort of this dataset consists of 17 patients with unilateral congenital ear canal atresia and 17 age- and gender-paired controls. The patients are adults with contralateral normal hearing, had no hearing aid or successful ear canal surgery before age 12, and have sufficient understanding of the Swedish language. Subjects with a history of severe psychiatric illness or neurological disease, any associated syndrome (Goldenhaar, CHARGE, etc.), or metallic artifacts were excluded from the cohort. In twelve of the patients, the right ear is affected. Eight of the patients are female and nine are male. The patients were all recruited in the Stockholm region. The ethical permit was granted by the Swedish ethical board (Dnr 2012/1661-31/3). The clinical dataset was pre-processed with the standard pre-processing pipeline of MRtrix3 (30) to remove artifacts and geometric distortions, which in turn uses methods from FSL (23).

### 2.2. TractSeg

**Figure 1:**
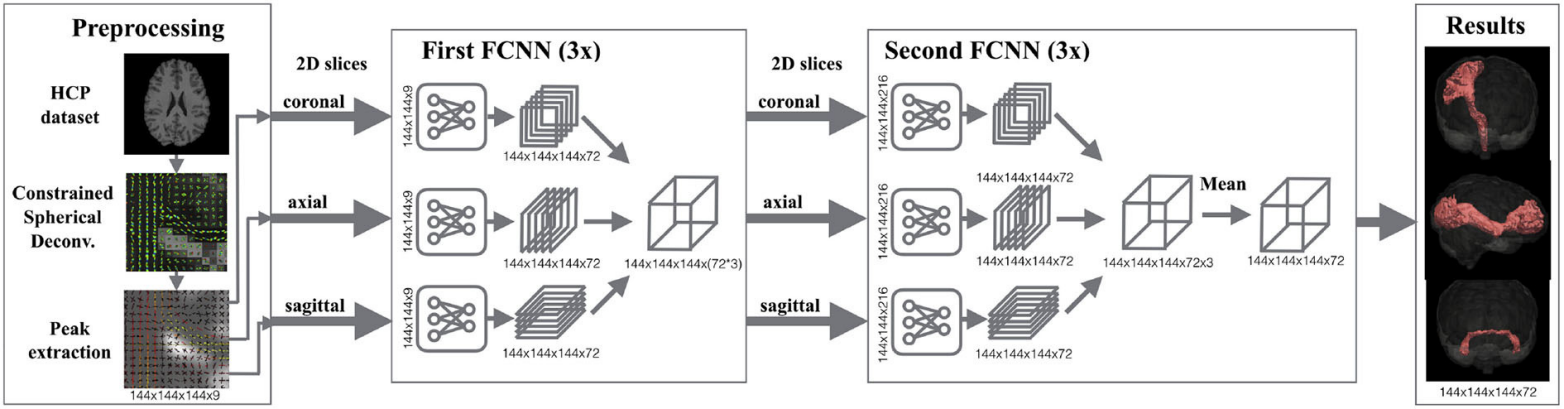
Segmentation pipeline of TractSeg. Left: The dMRI data is pre-processed for extracting the peaks of the fiber orientation distribution functions per voxel. These peaks are used as the input of the neural network. Middle: 2D U-Net-like fully convolutional neural networks (FCNNs) are trained to segment fiber bundles. Three networks are trained per axis (coronal, axial, sagittal) in two stages. While the goal in the first stage is to segment the fiber bundles using 2D information, the second stage aims at learning the best combination of the three intermediate results to generate the final segmentation. Right: Segmentation masks of 72 fiber bundles are generated. Figure reproduced from Wasserthal et al. (29), license CC BY 4.0.

TractSeg is a method that trains deep neural networks for segmenting fiber bundles (29). Figure 1 shows the pipeline of TractSeg. The steps of TractSeg are the following. First, the dMRI data must be pre-processed to remove artifacts and geometric distortions. Notice that this step is not required for HCP data since this dataset is already pre-processed (25). The clinical dataset was pre-processed with the tools provided in MRtrix3 (30). Second, fiber orientation distribution functions (fODF) are estimated per voxel using constrained spherical deconvolution (CSD) (31). The maxima (also known as peaks) of the fODFs can be seen as estimations of the most likely orientation fiber bundles in every voxel. Thus, the next step is to extract the largest peaks of the fODFs per voxel. Every peak is a vector whose direction and magnitude encode the most likely orientation of a fiber bundle and its strength, respectively. This strength, among many factors, is related to the density of fibers at the specific orientation of the peak. TractSeg assumes that a maximum of three fiber bundles can traverse a voxel. Thus, only the three largest peaks are input to the neural network. Notice that the magnitude of only one peak is not negligible in regions traversed by a single fiber bundle and two for those with two crossing fiber bundles. We used the option “–super_resolution” from TractSeg, which upsamples the peaks to an isotropic resolution of 1.25 mm.

Expert neuroanatomists manually segmented 72 different fiber bundles in 105 HCP subjects. These segmentations were used in TractSeg to train U-Net-like neural networks (32). As shown in Figure 1, TractSeg uses 2D neural networks (one per axis) in two stages. The first stage is used to generate masks of the fiber bundles by only considering the 2D information contained in the training slices. The second stage is used to learn the best combination to generate the final segmentation of the 72 fiber bundles. Notice that TractSeg uses a so-called 2.5D approach, that is, segmenting 3D structures with multiple 2D neural networks. Although it is possible to use 3D U-Nets instead, the authors argue that a 2.5D approach is more efficient and less prone to overfitting (29), which is in agreement with studies dealing with other segmentation problems [e.g., (33)].

TractSeg can be seen as a powerful method that can be used out-of-the-box to segment 72 fiber bundles (29). One of the main advantages of TractSeg is that, although it was trained on high-quality data [HCP young adult dataset (24, 25)], the neural network is also able to segment these bundles in dMRI data of clinical quality without any need for training. This is because the 72 targeted fiber bundles are relatively big. It is interesting to assess whether or not TractSeg can achieve the same performance with smaller fiber bundles, specifically the AR in clinical data. Thus, we generated training data for the AR from the same 105 HCP subjects used in TractSeg as described in the following section.

Although TractSeg does not include the core of the AR, it can be trained for that purpose (29). The training procedure requires the segmentation of the new fiber bundles of interest, ideally using the same dataset of the original article. Following the same approach of TractSeg, we used five-fold cross-validation with 105 subjects: 63 training subjects, 21 validation subjects, and 21 test subjects per fold. An additional set of 20 subjects was used for independent testing. As mentioned, newer versions of TractSeg have the option of using segmentation masks from XTRACT, including the AR. However, these segmentations consider not only the core but also can contain fiber bundles reaching the STG.

By design, TractSeg is able to segment fiber bundles beyond the original 72. For this, it is crucial to use high-quality segmentation masks of the new bundles during training. The following subsection describes the proposed methodology for generating such segmentation masks for AR.

### 2.3. Generation of training data

Probabilistic tractography (iFOD2) with anatomically-constrained tractography (ACT) (34) from MRtrix3 (30) was used for creating streamlines connecting the left HG to the left MGN and the right HG to the right MGN targeting the left and right AR, respectively. Masks of the HG and MGN at both hemispheres extracted with FreeSurfer (28) are available in the HCP database and were used as independent seeds for tractography. Thus, two sets of streamlines were obtained per side: one for streamlines starting at the HG and ending at the MGN and the other reversing the roles of two masks. We used the command “*tckgen*” in MRTrix3 (30) with the default parameters of iFOD2. Moreover, we used the options from ACT “*- backtrack*”, which tries to re-track partially truncated streamlines, and “*- crop_at_gmwmi*”, which crops the streamlines once they cross the boundary between gray and white matter.

**Figure 2:**
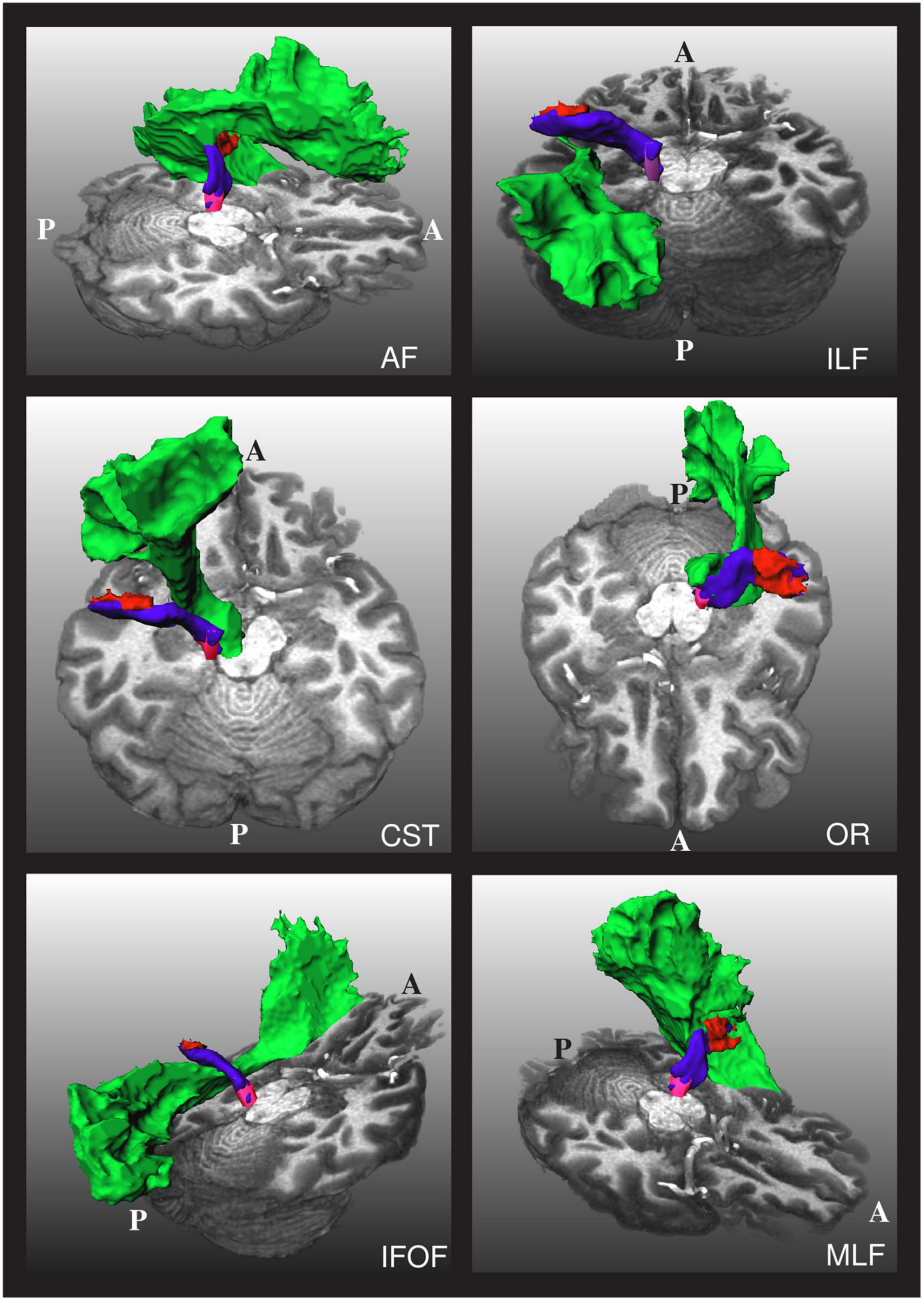
The relative position of the left acoustic radiation with six nearby fiber bundles for a subject of the human connectome project. The Heschl's gyrus, medial geniculate nucleus, and acoustic radiation of the left side of the brain are depicted in red, magenta, and blue, respectively. Each of the nearby fiber bundles is depicted in green, one per subfigure. A and P indicate the anterior and posterior sides of the brain, and T1w is used as a reference. The depicted acoustic radiation was computed using the methodology of Section 2.3.

As mentioned, one of the challenges in obtaining the AR is that it is very close to other fiber bundles, as shown in Figure 2. Our approach to tackling this issue is to reject any streamline reaching segmentation masks of nearby fiber bundles. In particular, we used the masks of the CST, IFOF, and ILF created by Wasserthal et al. (29) for training TractSeg to reject implausible AR streamlines.

As shown in Figure 2, the AF, OR, and MLF are too close to the AR that even some voxels can contain streamlines of different bundles. Thus, masks of AF, IR, and MLF cannot be used to reject implausible AR streamlines. Instead, we removed the voxels from these masks that are closer than 4 cm from both the HG and the MGN and used them to reject implausible AR streamlines. With this procedure, streamlines are allowed to enter the voxels close to the MGN and HG, which are also covered by the AF, OR, and MLF segmentation masks.

**Figure 3:**
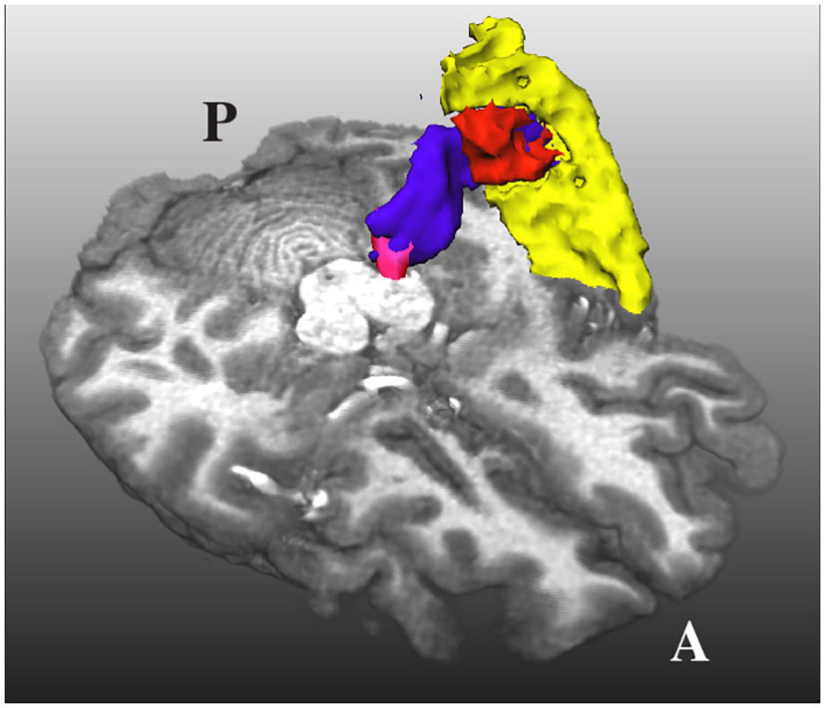
The acoustic radiation (in blue) from the medial geniculate nucleus (in magenta) and the Heschl's gyrus (in red) is also very close to the superior temporal gyrus (in yellow). A and P indicate the anterior and posterior sides of the brain, and T1w is used as a reference.

An additional problem is that the HG and the superior temporal gyrus (STG) are very close to each other, as shown in Figure 3. Due to the closeness between the HG and the STG, some streamlines can leak to the latter, especially when the MGN is used as the origin of the streamlines. In order to avoid this from happening, we used the mask of the STG extracted with FreeSurfer, which is available in the HCP database, to reject streamlines not ending in the HG. This step is crucial to remove possible streamlines not belonging to the core of the AR.

Notice that the described restrictions for generating streamlines are stringent and make the generation of training data computationally expensive. Actually, around 150,000 generated streamlines were discarded per every single accepted one. Thus, as stopping criteria, we set a maximum of 1,000 accepted streamlines, or 150 million generated streamlines in total per seed mask. The maximum length of each streamline was set to 60 mm. The two sets of streamlines per side were combined into a single tractogram. This procedure resulted in tractograms of at least 1,000 streamlines per side of the brain. Finally, a mask of the AR per side was created with the voxels traversed by at least ten streamlines. This procedure was successful in all HCP subjects.

It is important to emphasize that the original article of TractSeg (29) used whole-brain tractograms, each with 10 million streamlines with lengths between 40 and 250 mm. From these streamlines, only a few were part of the AR (fewer than 20 in all cases), which are not enough to generate reliable segmentation masks. The proposed procedure for generating streamlines of the core of the AR is expensive but effective for generating the masks that were used for training TractSeg.

## 3. Results

This section shows the results of the proposed methodology for segmenting the core of the AR applied to HCP data and the diffusion data acquired in a clinical setting on 17 patients with unilateral congenital ear canal atresia and 17 age- and gender-paired controls.

### 3.1. High-quality diffusion data

**Figure 4:**
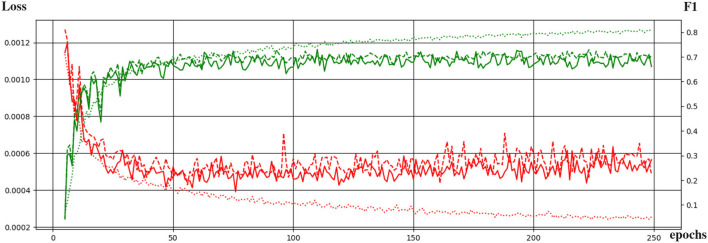
Evolution of the training of the neural network with the training epochs. The loss function and the F1 score are shown in red and green, respectively. Dotted, continuous, and dashed lines correspond to performance during training, validation, and testing.

Figure 4 shows the curves of the F1 score during validation and testing on HCP data. The best performing network attained an F1 score of 0.73 during testing. The F1 score is equivalent to the Dice score for segmentation purposes.

**Figure 5:**
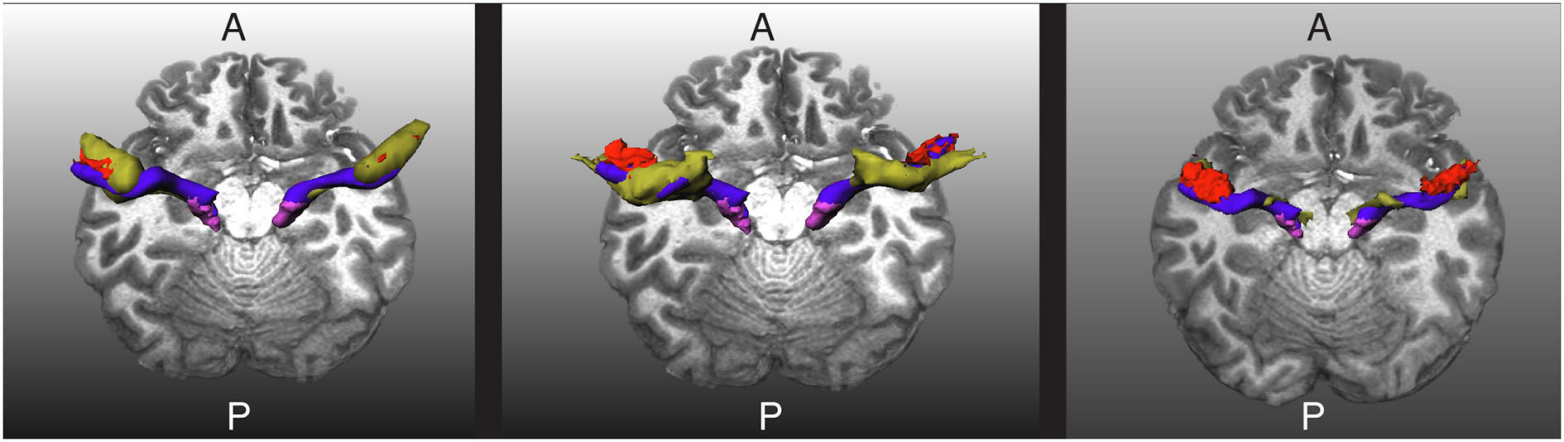
Visualization of the extracted acoustic radiation for one subject from the human connectome project in blue. The Heschl's gyrus and medial geniculate nucleus are depicted in red and magenta, respectively. Left: The atlas from Bürgel et al. (2) is shown as a reference in yellow. Middle: The atlas from Maffei et al. (16) is shown as a reference in yellow. Right: The segmentation obtained with XTRACT (20) is shown in yellow as a reference. A and P indicate the anterior and posterior sides of the brain, and T1w is used as a reference.

**Figure 6:**
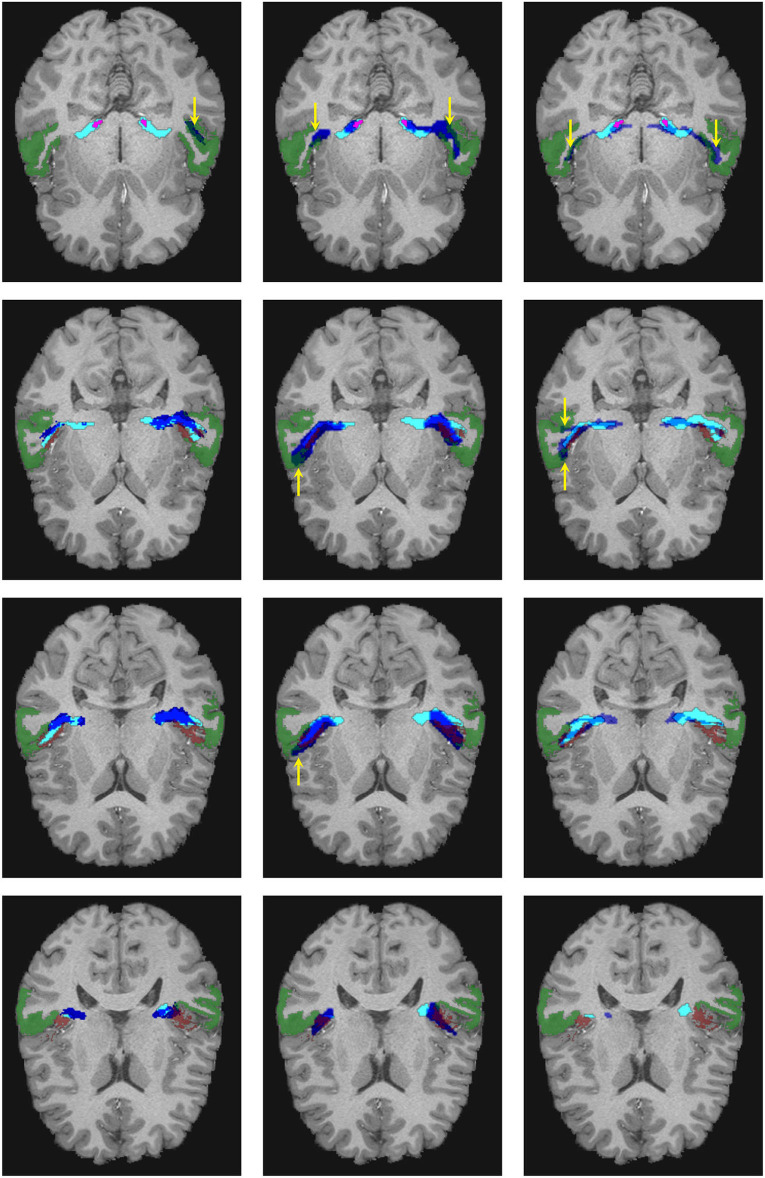
Visual comparison of the segmentation masks in one subject of the human connectome project. First column: Segmentation mask of the proposed methodology (in cyan) and the atlas by Maffei et al. (16) (in blue). Second column: Segmentation mask of the proposed methodology (in cyan) and the atlas by Bürgel et al. (2) (in blue). Third column: Segmentation mask of the proposed methodology (in cyan) vs. the result from XTRACT (in blue). Every row corresponds to a different axial slice. The superior temporal gyrus (STG), medial geniculate nucleus, and Heschl's gyrus are depicted in green, magenta, and brown, respectively. Yellow arrows indicate where the segmentation masks reach the STG.

We tested the trained network in 20 additional HCP subjects not used for training. As shown in Figures 5, 6 for one of these subjects, the segmentation results of the core of the AR at both sides are anatomically plausible. From the figure, it can be seen that there are differences between atlases. The segmentation generated from our methodology is more conservative than the atlases and XTRACT. For example, the generated segmentation masks always stop at the boundary between white matter and the HG, while, e.g., (2) usually overlaps with the HG and is more likely to reach the STG. Most of the generated masks of AR overlap with the two atlases and XTRACT.

As shown in Figure 6, the atlases and XTRACT tend to reach regions of the STG (see yellow arrows), sometimes in regions not adjacent to the HG. It can also be seen that the segmentation masks differ from each other, especially in the region close to the HG.

Using visual inspection, we found that the proposed methodology was able to extract anatomically plausible AR in all 20 subjects used for independent testing.

### 3.2. Diffusion data acquired in a clinical setting

We applied the trained network on dMRI data of 17 subjects with unilateral ear canal atresia and 17 controls. As mentioned, these images were acquired in a clinical setting (*b* = 1, 000*s*/*mm*^2^, 60 directions, spatial resolution = 2.3 *mm* isotropic). This case is more challenging than the segmentation of the HCP data due to the low spatial and angular resolution and the relatively low *b*-value used in the acquisition. Table 1 shows the number of cores of the ARs that were completely reconstructed, were reconstructed in fragments, or where the method failed. As shown, the method was able to completely reconstruct the core of the AR in most cases (53/68 = 77.9%) with a similar performance between patients and controls (24 vs. 29). The method yielded fragmented cores of the ARs in 14 cases (20.5%) and more often in patients than in controls (9 vs. 5). The fragments were visually inspected. In most cases, the core of the AR was fragmented into two pieces, each of them closer to either the MGN or the HG. In a few cases, the core of the AR appeared as a blob in the middle between the MGN and the HG. In the 14 cases, the fragments were always located at the region where the AR is expected to be. The method only failed to reconstruct the left AR of a single patient. The trained network was also more consistent in yielding uncut segmentations on the left side (2 cases on the left vs. 12 on the right).

**Table 1:** The number of subjects in which the proposed methodology was able to reconstruct the complete acoustic radiation (AR) (Uncut), split the AR into fragments (Fragm.), or completely failed (Fail) per side in the clinical dataset of unilateral ear canal atresia.

	**Left AR**			**Right AR**			**ARs of both sides**		
	**Uncut**	**Fragm**.	**Fail**	**Uncut**	**Fragm**.	**Fail**	**Uncut**	**Fragm**.	**Fail**
Patients R (*N* = 12)	10	1	1	8	4	0	29	5	1
Patients L (*N* = 5)	4	1	0	2	3	0	6	4	0
All Patients (*N* = 17)	14	2	1	10	7	0	24	9	1
Controls (*N* = 17)	17	0	0	12	5	0	29	5	0
All subjects (*N* = 34)	31	2	1	22	12	0	53	14	1

Patients R and Patients L indicate the side of the affected ear.

**Figure 7:**
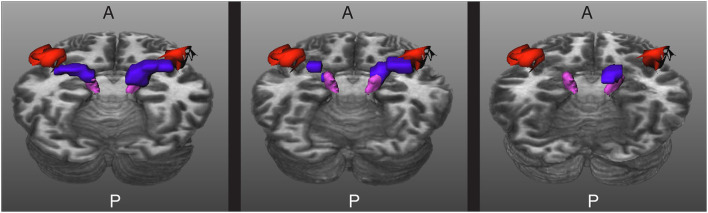
Results for three images acquired in a clinical setting. The core of the acoustic radiations (ARs) are depicted in blue, the Heschl's gyrus (HG) in red, and the medial geniculate nucleus (MGN) in magenta. Left: The core of the ARs are completely extracted. Middle: The core of the ARs are fragmented into two pieces. Right: The method gave a blob in between the MGN and the HG for the right side and was unable to segment the core of the AR of the left side. A and P indicate the anterior and posterior sides of the brain, and T1w is used as a reference.

In the cases where TractSeg was not able to extract the complete core of the AR, it is possible to use the masks to guide tractography. For this, not only the MGN and the HG are used as seed regions, but also the results of the segmentation with TractSeg. This makes it more likely for tractography to compute streamlines that comply with the strict restrictions described in Section 2.3. Figure 7 shows the results obtained for some of the subjects.

**Figure 8:**
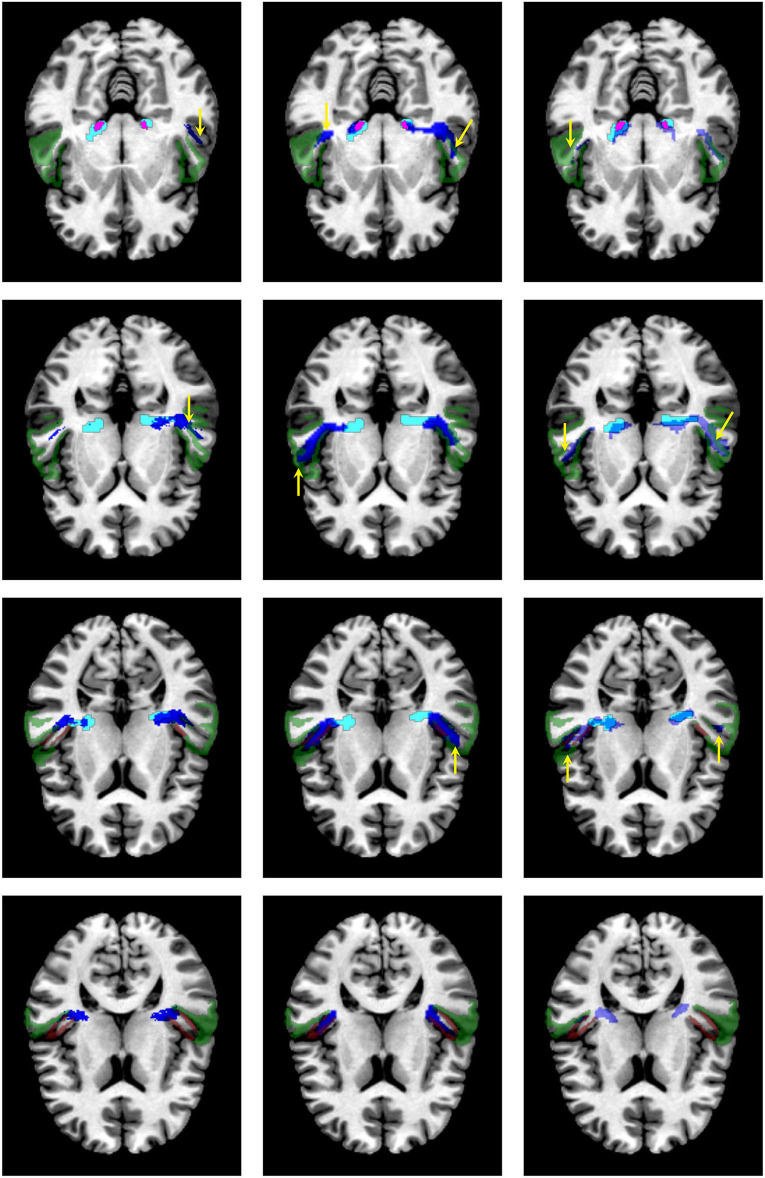
Visual comparison of the segmentation masks on one subject of the clinical dataset. First column: segmentation mask of the proposed methodology (in cyan) and the atlas by Maffei et al. (16) (in blue). Second column. segmentation mask of the proposed methodology (in cyan) and the atlas by Bürgel et al. (2) (in blue). Third column: segmentation mask of the proposed methodology (in cyan) vs. the result from XTRACT (in blue). Every row corresponds to a different axial slice. The superior temporal gyrus (STG), medial geniculate nucleus, and Heschl's gyrus are depicted in green, magenta, and brown, respectively. Yellow arrows indicate where the segmentation masks reach the STG.

Figure 8 shows a visual comparison of the segmentation masks obtained with the proposed methodology, the atlases by Bürgel et al. (2) and Maffei et al. (16), and XTRACT for one subject from the clinical dataset where the methodology was able to extract the core of the AR. As shown, the atlases and XTRACT tend to reach more the STG. Except for the atlas by Bürgel et al. (2), the other methods have problems entering the cavity of the HG in this specific subject.

**Figure 9:**
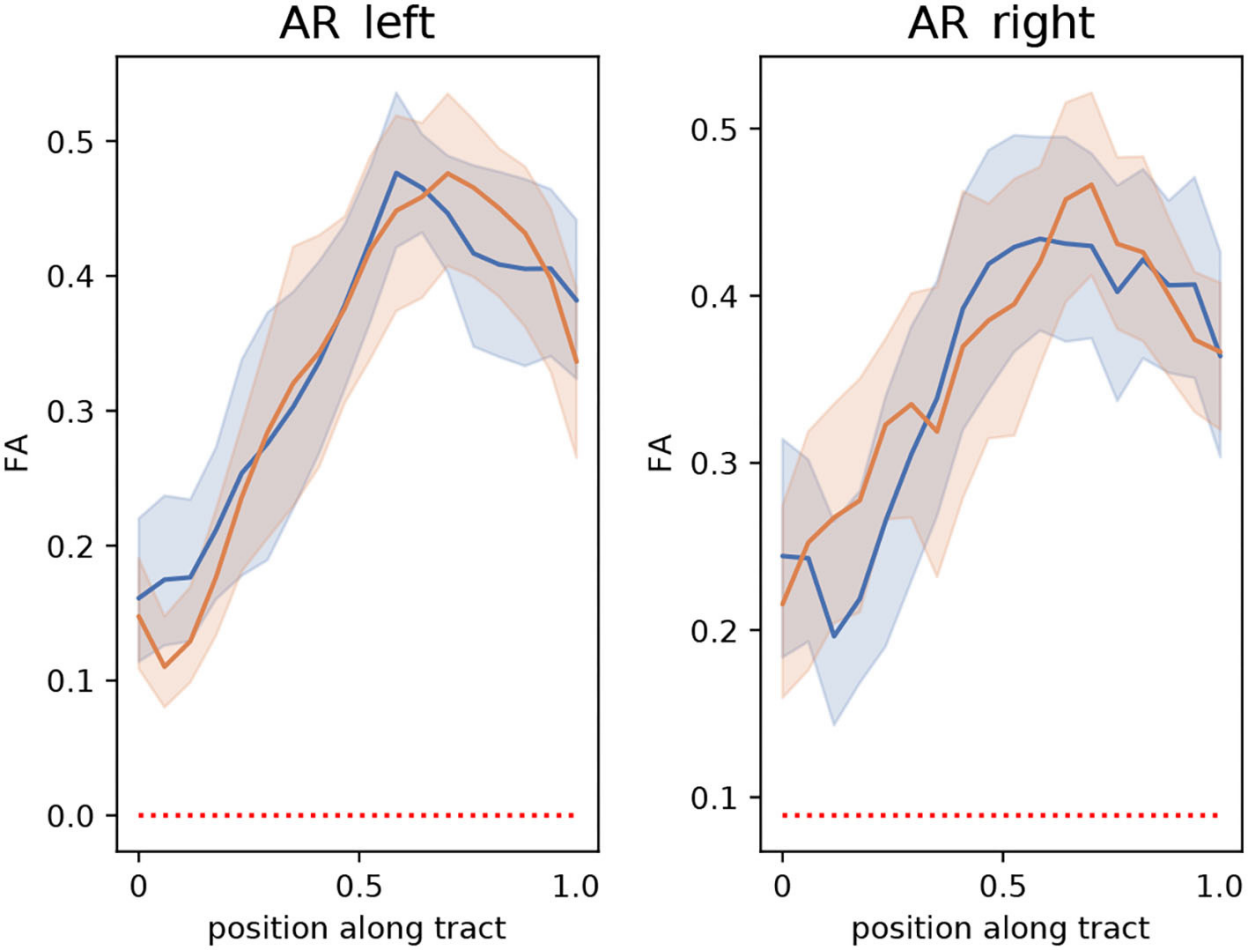
Bundle analysis of the fractional anisotropy (FA) applied to the left and right acoustic radiations (AR) for the clinical dataset used in this paper. The mean FA of patients and controls along the tracts are shown with lines in blue and orange, respectively. The 95% CIs are shown in light blue and light orange bands, respectively for the two groups. Position 0 and 1 along the tract are located at the medial geniculate nucleus and the Heschl's gyrus, respectively.

The extracted segmentation masks can be used for different group analyses. Among many other options, one can use the masks to restrict tractography and perform bundle analytics (35). To showcase this application, we used the implementation of TractSeg for bundle analytics. In brief, the method runs tractography, but unlike the procedure described in Section 2.3, the generated streamlines are only restricted to traversing the segmentation mask of the AR. Using the AR masks is much less restrictive than using the neighboring fiber bundle masks and, thus, is much less time-consuming (ca. 10–20 min. per subject). Then, the generated streamlines are used to sample the maps of fractional anisotropy (FA) or any other measurement along the path of the streamlines. This way, it is possible to assess differences between the groups along the trajectory of the AR. Figure 9 shows a bundle analysis of the FA applied to the AR for the clinical dataset. As shown, the FA starts at a very low value at the MGN, goes up in the middle, and down again to the end close to the Heschl's gyrus. It can be seen that the 95% CIs (shown with colored bands) are relatively large. In fact, these CI were 2–3 times larger than for the cortical spinal tract (CST) and other large tracts. This could mean that the intersubject variability is higher for the AR than for large fiber bundles. We performed *t*-tests along the tract that were corrected for multiple comparisons to account for family-wise errors. With this procedure, we did not find any statistically significant difference between the two groups at any point along the tract.

## 4. Discussion

Previous studies have shown that extracting the AR is possible *in vivo* on data from the MGH adult diffusion dataset of HCP with ultra-high *b*-values up to 10,000 *s*/*mm*^2^ (16). In this study, we showed that extracting the core of the AR in high-quality dMRI data with lower *b*-values (*b* = 1,000, 2,000, and 3,000 *s*/*mm*^2^) from the HCP young adult dataset by using masks of neighboring fiber bundles is also possible. One issue of our approach is that our strategy is very restrictive and time-consuming.

Thus, in order to reduce the computation time, we trained the neural network of TractSeg (29) with the segmentation masks of the core of the AR created from HCP data. There are two main advantages of using TractSeg for segmenting the AR compared to using atlases: (a) that the resulting masks are subject-specific, and (b) it is not necessary to do registration to a template. Regarding the former, subject-specific masks can tackle the anatomical variability of the AR, HG, and MGN. As for the latter, misregistrations can generate errors that are not a problem for TractSeg. An alternative to using TractSeg is to generate the core of the AR as proposed in Section 2.3. The main gain of using TractSeg is that the segmentation mask is obtained in a few seconds instead of several hours of the proposed methodology from Section 2.3.

The trained neural network of TractSeg was able to segment the core of the AR in HCP data in a few seconds instead of several hours. We used a workstation equipped with an Intel Xeon CPU E5-2630 v3 with 8 cores at 2.40 GHz, and a GPU NVIDIA GeForce GTX 1070. The processing of one HCP subject using the methodology described in Section 2.3 was 8–10 h in this workstation. Computing the peaks of the fODFs took approximately 1 min and applying the trained neural network took around 40 s for both the HCP data and the clinical data. The segmentations generated by the trained neural network were anatomically plausible when applied to an independent set of subjects from HCP. The methodology proposed in Section 2.3 is conservative. Thus, the segmentation masks obtained with the neural network are also conservative compared to the publicly available atlases of the AR. We argue that it is important to have a conservative approach to extracting the core of AR. This way, the downstream conclusions drawn from group analyses of the AR will become more meaningful.

The trained neural network had more problems with data acquired in a clinical setting. Still, it was able to completely segment the core of the ARs in 77.9% of the cases, yielded fragmented masks in 20.6% of the cases, and only failed in a single subject. The performance was very similar in patients and controls. The neural network tended to reconstruct the core of the left AR better than the core of the right AR.

As shown in some cases, the neural network yields a fragmented segmentation. Such fragments can be used as seeds for tractography, which has the advantage of reducing the high cost of running tractography to extract the core of the AR.

We compared the proposed methodology with the segmentation generated by TractSeg (29) trained with masks created with XTRACT (20, 21). From the results, an important difference between our methodology and XTRACT is that the latter included tracts that reached the STG in the segmentation masks. It is important to differentiate the fibers connecting only the MGN and the HG from those that can get the STG, as they can have different purposes in the human brain (9). For example, Ito et al. (36) reported that the STG might be involved in the joint processing of visual and auditory stimuli. Unlike XTRACT, the proposed methodology actively removes the fibers reaching the STG to target the core of the AR. At this stage, it is not possible to know if the fibers covered by XTRACT and not covered by our methodology belong to the belt of the AR. The STG is a structure that is larger compared to the HG. Thus, it is not clear which substructures of the STG might be part of the AR. Such information is crucial to assess whether the voxels reaching the STG by the masks of XTRACT belong to the AR or are artifacts.

Unlike our methodology, XTRACT was able to generate the AR in all cases. Since XTRACT uses less restrictive rules for generating the masks, they cover more voxels, which makes TractSeg increase its robustness at the cost of being less specific. In some cases, the XTRACT masks covered parts of the ventricles and the most posterior parts of the STG, almost reaching the medial temporal gyrus. Thus, we recommend a manual review of these masks before any further analysis.

Previously, Bertó et al. (37) added prior information for improving the segmentation of fiber bundles. Our results are in line with that study since we show that adding the segmentation masks of other bundles is needed for the segmentation of small fiber bundles like the AR.

We showcased the use of segmentation masks by performing a bundle analysis on the clinical dataset to assess differences in FA between patients and control in the AR. We did not find any statistically significant difference between the groups. The 95% CI was larger than other bundles (e.g., the CST). This suggests that the intersubject variability is higher for the AR.

The results of this study are encouraging but also show that more research is needed toward a fully automatic segmentation of the AR from images acquired in clinical settings. For example, as mentioned, TractSeg uses three peaks of the fODFs (29). Recently, it has been argued that up to seven fiber bundles might appear in certain brain regions (38). Thus, it is possible that more peaks could be helpful for extracting the AR. However, enlarging the number of inputs to the neural network has the disadvantage of needing more training data or changing the neural network architecture, which is beyond the scope of this article. Although TractSeg (29) can still be considered state-of-the-art for fiber bundle segmentation, new AI-based segmentation methods have recently been proposed [e.g., (39–42)]. It is interesting to assess if adapting these methods can yield better results for segmenting the AR. Plans for the future also include the analysis of the AR for other diseases affecting the auditory system and datasets acquired in different clinical settings.

This study has many limitations. One of the main issues is that there is not possible to have a personalized ground truth that can be used to assess the accuracy. This is a general limitation of any method based on tractography. The atlas by Bürgel et al. (2) was created from histology and is expected to depict the anatomy of AR better. However, the variability of the HG, MGN, and the AR among subjects, makes it less appropriate for group analyses. A second limitation is that although FreeSurfer is relatively accurate for segmenting the HG [Desikan et al. (43) reported intraclass correlations between automatic and manual segmentations of 0.712 and 0.719 for the left and right HG, respectively], it can be inaccurate in cases where the HG has duplications. Marie et al. (44) found in a cohort with 430 participants that 36.6 and 48.8% of the right-handed subjects and 30.8 and 39.4% of the left-handed subjects had duplications on the left and right side, respectively. Considering duplications of the HG in the pipeline is clinically relevant since they have been associated with neurological conditions (45). In order to account for this anatomical variability of the HG, it would be necessary not only to use during training more accurate segmentation tools tailored explicitly for the HG [e.g., TASH (46)] but also to train independent TractSeg models for subjects with and without duplications in the HG. The most appropriate TractSeg model for a specific subject could be chosen once the type of HG is detected. Still, it is uncertain whether such an approach could lead to differences in AR.

## 5. Conclusion

In this study, we proposed a methodology to extract the core of the AR in subjects from the HCP young adult dataset by using masks of neighboring fiber bundles obtained with TractSeg. Since the procedure is expensive, we trained TractSeg to extract the AR automatically. For this, we used the masks of the AR extracted from a set of subjects from the HCP young adult dataset. The trained neural network was applied both to unseen subjects of the HCP young adult dataset and a clinical dataset.

The main conclusion of this study is that it is possible to segment the core of the AR in most cases, even in images acquired in clinical settings in a few seconds with the trained network. In case it is not possible to reconstruct the core of the AR, the results can be used as masks for tractography.

## Data availability statement

The data analyzed in this study is subject to the following licenses/restrictions: We used data from the Human Connectome Project (HCP), WU-Minn Consortium (Principal Investigators: David Van Essen and Kamil Ugurbil; 1U54MH091657) funded by the 16 NIH Institutes and Centers that support the NIH Blueprint for Neuroscience Research; and by the McDonnell Center for Systems Neuroscience at Washington University. The training data and the trained neural network is available at https://doi.org/10.5281/zenodo.7052849. The data from the Karolinska Institute cannot be shared. Requests to access these datasets should be directed to RM, rodmore@kth.se.

## Ethics statement

The Human Connnectome Project (HCP) data is publicly available and all authors have accepted the HCP Open Access Data Use Terms. The acquisition of the dataset from Karolinska Institute was reviewed and approved by the Swedish Ethical Board (Etikprövningsmyndigheten) Dnr 2012/1661-31/3. The patients/participants provided their written informed consent to participate in this study.

## Author contributions

MS: conceptualization, data curation, investigation, methodology, and writing—review and editing. CE: conceptualization, methodology, resources, writing—review and editing, supervision, and funding acquisition. RM: conceptualization, methodology, visualization, resources, project administration, writing—original draft, review and editing, supervision, and funding acquisition. All authors contributed to the article and approved the submitted version.

## Funding

This study was partially supported by VINNOVA, through AIDA, the Center for Innovative Medicine (CIMED), Region Stockholm, and Digital Futures, Project dBrain.

## Conflict of interest

The authors declare that the research was conducted in the absence of any commercial or financial relationships that could be construed as a potential conflict of interest.

## Publisher's note

All claims expressed in this article are solely those of the authors and do not necessarily represent those of their affiliated organizations, or those of the publisher, the editors and the reviewers. Any product that may be evaluated in this article, or claim that may be made by its manufacturer, is not guaranteed or endorsed by the publisher.

## Footnotes

^1^ https://www.humanconnectome.org/hcp-protocols
